# Oliver Wendell Holmes

**Published:** 1887-10-08

**Authors:** 


### Oliver Wendell Holmes.

By Dr. Caterina.

(*Continued from p. 6.*)

Dr. Holmes is tender to all humanity, balancing sin and failure against hindrance and temptation. Conversation at the breakfast-table has turned on drunkenness, and this is how the Autocrat explains the vice:—

"Our brains are seventy-year clocks. The Angel of Life winds them up once for all, then closes the case, and gives the key into the hand of the Angel of the Resurrection. Tic-tac! tic-tac! go the wheels of thought; our will cannot stop them, they cannot stop themselves; sleep cannot still them; madness only makes them go faster. Unless the will maintain a certain control over these movements, which it cannot stop, but can, to some extent, regulate, men are very apt to try to get at the machine by some indirect system of leverage or other. They clap on the brake by means of opium; they change the maddening monotony of the rhythm by means of fermented liquors. It is because the brain is locked up, and we cannot touch its movements directly, that we thrust these coarse tools through any crevice by which they may reach the interior, and so alter its rate of going for a while, and at last spoil the machine. Men who exercise chiefly those faculties of the mind which work independently of the will—poets and artists, for instance, who follow their imagination in their creative moments instead of keeping it in hand, as your logicians and practical men do with their reasoning faculty—such men are too apt to call in the mechanical appliances to help them to govern their intellect."

"He means they get drunk," interprets the young man named John, who is one of the Autocrat's most delightful fellow-boarders—a good-natured youth, slangy and flippant in speech, but honest-hearted and shrewd-brained—a Yankee of the Yankees, with a good deal more native common-sense and incomparably more real refinement of feeling than the pseudo-cultured American gentlemen of Mr. Howells, who are guilty of the most arrant snobbery to show that they are not used to hearing provincialisms in speech nor to seeing provincialism in dress. Perhaps the different impression made upon us by the two authors may be accounted for by the fact that the Doctor is so sure of himself, thanks to generations of hereditary culture, that he does not need to thrust upon his readers his knowledge of the ways of society. He appreciates at its true value this hereditary habit of refinement, as most Americans do; and admits the fact straightforwardly, as many of them do not. It is to be feared that the Autocrat has not that respect for self-made men which, as a rule, they somewhat aggressively demand.

"Self-made men?" he says. "Well, yes. Of course everybody likes and respects self-made men. It is a great deal better to be made in that way than not to be made at all. Your self-made man, whittled into shape with his own jack-knife, deserves more credit, if that is all, than the regular engine-turned article, shaped by the most approved pattern, and French-polished by society and travel. But as to saying that one is every way the equal of the other, that is another matter."

This opinion, though more courteously expressed, is probably somewhat akin in meaning to the dictum of a canny Scot, who remarked that when a man claimed to be self-made, he "took a great responsibility off the Almighty."

Be it observed that it is the man whose self-manufacture tends to the accumulation of material riches, whom Dr. Holmes does not care to have too much association with. The nobler self-made man, who against all disadvantages of circumstance has acquired mental wealth and developed his nobler nature, has his deepest respect, and the praise he gives to a brother poet is that he is

"Crowned with the noblest wreath of rhyme,
The holly-leaf of Ayrshire's peasant."

The honour paid to the self-made millionaire is too often of a toadying nature, and is of the nature of humbug, a thing which medical men as a rule despise. Perhaps no remark Dr. Holmes ever made has so shocked amiable, but illogical people (who are as a rule the most liable to suffer from the malignant types of humbug), as that utterance of the Professor, that the piety occasionally seen in young children is usually pathological in its nature—due, that is, to morbid conditions, and not characteristic of healthy youth. John Bunyan knew the same truth—"the more healthy the man is, the more prone he is to evil," he tells us; but it is to be feared that he enunciates the axiom rather to the disparagement of bodily health than to the depreciation of the piety engendered by disease.

The narrowing influence of theology of this sort is a thing our Autocrat cannot bear.

"We must get over the habit of transferring the limitations of the nervous temperament and of hectic constitutions to the great source of all the mighty forces of nature, animate and inanimate. We may confidently trust that we have over us a Being thoroughly robust and grandly magnanimous, in distinction to the Infinite Invalid bred in the studies of monomaniacs, who corresponds to a very common human type, but makes us blush for him when we contrast him with a truly noble man."

Yet for those who suffer most from this monomania he has a sympathy that is mingled with respect.

"Insanity is often the logic of an accurate mind overtasked. Good mental machinery ought to break its own wheels and levers, if anything is thrust among them suddenly which tends to stop them or reverse their motion. A weak mind does not accumulate force enough to hurt itself; stupidity often saves a man from going mad. We frequently see persons in insane hospitals, sent there in consequence of what are called religious mental disturbances. I confess that I think better of them than of many who hold the same notions, and keep their wits, and appear to enjoy life very well outside of the asylums."

It is to be feared that Dr. Holmes would look upon the people who are capable of holding theological views which would condemn a great number of their fellow-mortals to endless agonies, and don't go mad with the pity and horror of it, as specimens of what he calls moral teratology. Teratology, it may be well to mention, is that branch of biological science which deals with the monstrosities and malformations which are sometimes seen in animals. The bearded lady and the two-headed nightingale, at whom the multitude gaze wonderingly, with a pleasing cold thrill going down their spine, and giving them much the same satisfaction as a vanilla ice on a hot day, mingled with a certain flavour of conceited thankfulness that they themselves are not as the curiosities on the platform—these are specimens of teratology; along with the two-headed calf, and the eccentric chicken which was hatched without a head at all. Strange as these products of nature are, they exist in obedience to fixed rules, which in minor instances can be traced according to the law of heredity, and can even, if one may so speak, be manufactured to order, if one should want them.

Similarly, in the world of thought, there are ideas cherished by otherwise good and sensible people, which would seem incredible, monstrous, in their selfishness and cruelty, if one could not see the origin of them in the environment in which those who hold them are placed, and the ideas they have inherited from ancestors more or less direct. Heredity and environment, much as the terms have been misapplied and mocked at, are the true keys to the mysteries of nature. The strong ones of the human race are those who conquer their environment; the weak are those whose environment conquers them, who are the perpetual victims of circumstances over which they have no control. The first are they who build and draw the car of progress; the second act, not altogether uselessly sometimes, as a drag on the wheel.

*(To be continued.)*

